# Epidémiologie et étiologies des insuffisances cardiaques à Lomé

**DOI:** 10.11604/pamj.2014.18.183.3983

**Published:** 2014-06-25

**Authors:** Machihudé Pio, Yaovi Afassinou, Soulemane Pessinaba, Soodougoua Baragou, Jacques N'djao, Borgatia Atta, Efadzi Ehlan, Findibé Damorou, Edem Goeh-Akué

**Affiliations:** 1 Service de Cardiologie, Centre Hospitalier Universitaire Sylvanus Olympio de Lomé, Lomé, Togo

**Keywords:** Insuffisance cardiaque, épidémiologie, étiologie, Togo, heart failure, epidemiologie, etiology, Togo

## Abstract

**Introduction:**

L'objectif est de déterminer la fréquence et les principales étiologies des insuffisances cardiaques (IC) en milieu cardiologique à Lomé.

**Méthodes:**

Il s'agissait d'une étude transversale réalisée de janvier 2010 au Janvier 2012 dans le service de cardiologie du CHU Sylvanus Olympio de Lomé. Elle a porté sur 297 dossiers des patients hospitalisés pour IC. L’échodoppler cardiaque était indispensable pour l'inclusion du dossier dans l’étude.

**Résultats:**

L'IC représentait 25,6% des hospitalisations. L’âge moyen des patients était de 52,2 ± 16,7 ans avec un sex ratio à 0,93. Le tableau clinique était celui d'une IC globale (67%), IC gauche (31,3%) et IC droite (1,7%). La fraction d’éjection du ventricule gauche (FEVG) moyenne était de 0,4 ± 0,17 (extrêmes 0,07 et 0,88). Les diagnostics lésionnels des IC étaient les cardiomyopathies dilatées (60%), l'IC à FEVG conservée (11,4%), les valvulopathies (11,8%). Les étiologies étaient l'hypertension artérielle (HTA) (43,1%), les coronaropathies (19,2%), la cardiomyopathie du péripartum (11,8%), les valvulopathies (11,8%), la cardiothyréose (3%), le c'ur pulmonaire chronique (2,7%), les cardiopathies congénitales (2,7%), l'alcoolisme chronique (2%), le VIH (3,4%) et idiopathique (5,9%).

**Conclusion:**

L'IC est fréquente à Lomé et touche les sujets jeunes. Les lésions sont sévères et avancées sous forme de cardiomyopathies dilatées.

## Introduction

L'insuffisance cardiaque (IC) constitue un important problème de santé publique par sa fréquence, sa mortalité, mais aussi par sa morbidité et, les ressources médicales et économiques significatives qu'elle absorbe [1–3]. En occident l'IC est de loin l'affection la plus fréquente, la plus coûteuse et de pronostic réservé [4]. En Afrique l'IC constitue la principale circonstance de découverte des maladies cardiovasculaires [5, 6]. Les objectifs de ce travail étaient de déterminer la prévalence, les diagnostics lésionnels et les étiologies de l'IC en hospitalisation cardiologique à Lomé.

## Méthodes

L’étude s'est déroulée dans le service de cardiologie du CHU Sylvanus Olympio (SO) de Lomé qui constitue le 1er centre national de référence. Il s'agit d'une étude transversale descriptive menée sur une période de 24 mois (janvier 2010 à janvier 2012). Etaient inclus dans l’étude les patients des deux sexes hospitalisés pour IC affirmée sur les bases cliniques, radiographiques, électrocardiographiques et confirmée par une échocardiographie doppler. Les critères diagnostiques de l'IC utilisés dans cette étude étaient ceux de Framingham et de la Société Européenne de Cardiologie [7, 8]. La dysfonction systolique a été définie par une fraction d’éjection inférieure à 50%. Les éléments épidémiologiques étudiés étaient: l’état civil, la profession, la situation matrimoniale et les conditions socio-économiques. Nous avons analysé: les facteurs de risque cardiovasculaire, les antécédents (ATCD) cardiovasculaires, les signes fonctionnels, les motifs d'admission, le mode de survenu de l'IC et les données de l'examen physique en particulier l'examen cardiovasculaire.

L'histoire de la maladie associée aux ATCD, à la clinique et aux examens paracliniques ont permis de poser le diagnostic des cardiomyopathies dilatées (CMD) hypertensive, ischémique, éthylique, inflammatoire, toxique et anémique. La cardiomyopathie du péripartum (CMPP) était retenue chez les femmes présentant une IC entre le huitième mois de grossesse et les cinq premiers mois du post-partum sans étiologie retrouvée. Le diagnostic d'une valvulopathie organique était posé devant les signes d'IC, la présence d'un souffle cardiaque et les données de l’échodoppler cardiaque. Nous avons retenu le diagnostic de cardiopathie ischémique devant l'association d'une histoire clinique avec des précordialgies ou non, des troubles de la repolarisation péjoratifs à l'ECG, les valeurs de la troponine I, et les troubles segmentaires de la contractilité pariétale à l’échocardiographie doppler. Le diagnostic de cardiothyréose a été retenu devant des signes cliniques de thyrotoxicose, des signes de dysfonction myocardique à l’échodoppler cardiaque, les taux de FT3 et FT4 élevées et le taux bas de la TSH. Le diagnostic des cardiopathies congénitales et de coeur pulmonaire chronique (CPC) étaient posés devant les ATCD, les signes cliniques et les données de l’échodoppler cardiaque. Le diagnostic des IC à fraction d’éjection conservée était posé devant les signes d'IC gauche avec une dysfonction diastolique et une fraction d’éjection conservée. Le diagnostic de CMD idiopathique était retenu en l'absence de facteurs pouvant expliquer la dilatation des cavités cardiaques et la dysfonction myocardique

Nos données ont été analysées à l'aide du logiciel Epi Info version 3.5.3 du 26 Janvier 2011, version anglaise.

## Résultats

Pendant la période de l’étude nous avions eu 1296 hospitalisations. Les IC concernaient 297 patients soit 25,6% des hospitalisations. La moyenne d’âge était de 52,2 ans ± 16,7 (extrêmes 18ans et 106 ans). Le sex ratio était de 0,93.

L'HTA était le principal ATCD et concernait 52,5% des patients. L’éthylisme chronique et le tabagisme représentaient respectivement 30,6% et 9,1%. La dyspnée d'effort (75,1%) était le principal signe fonctionnel suivie des oedèmes des membres inférieurs (OMI) (46,9%). La dyspnée était de stade III dans 58,2% et stade IV chez 29,8% des patients. L'IC était globale chez 199 (67%) patients.

L'index cardio-thoracique (ICT) moyen était de 0,69 ± 0,06 (extrêmes de 0,48 et 0,93). La cardiomégalie était présente chez 248 patients (83,5%) parmi lesquels 72,8% avaient ICT ≥0,60.

Les principales anomalies électrocardiographiques étaient: un micro-voltage diffus (17,8%), la tachycardie sinusale (62,2%), une hypertrophie ventriculaire gauche (50,5%), une hypertrophie ventriculaire droite (12,6%), des extrasystoles ventriculaires (42,7%), des extrasystoles auriculaires (20,3%), une arythmie complète par fibrillation auriculaire (19,7%), une onde Q de nécrose (33,2%) et un bloc de branche gauche complet (19,9%).

Le Tableau 1 montre les résultats obtenus à l’échocardiographie Doppler. Le diamètre moyen du VG en diastole était de 61,3 ± 10,9 mm; celui de l'oreillette gauche de 46,8 ± 8,6mm. Chez 45,8% des patients, la FEVG était inférieure à 0,2. Le ventricule droit avait un diamètre moyen de 52,1 mm et l'oreillette un diamètre moyen de 44 mm. Les valvulopathies organiques étaient reparties comme suit: insuffisances mitrales 21 patients (60%), maladie mitrale 7 patients (20%), insuffisance aortique 4 patients (11,4%) et un rétrécissement aortique (8,6%). La pression artérielle pulmonaire systolique (PAPS) moyenne était de 74,4 ± 17 mm Hg.

**Tableau 1:** Résultants de l’échocardiographie Doppler des patients

Paramètres	Effectif	Pourcentage (%)
Dilatation du ventricule gauche	210	70,7
Dilatation de l'oreillette gauche	175	59
Dilatation du ventricule droit	63	21,2
Dilatation de l'oreillette droite	67	21,6
Dysfonction systolique du ventricule gauche	216	79,1
Anomalies de la relaxation	95	32
Anomalies de la compliance	177	59,5
Valvulopathies	35	11,8
Hypertension artérielle pulmonaire	137	46,1
Thrombus intra cavitaire	21	7,1

**Figure 1:**
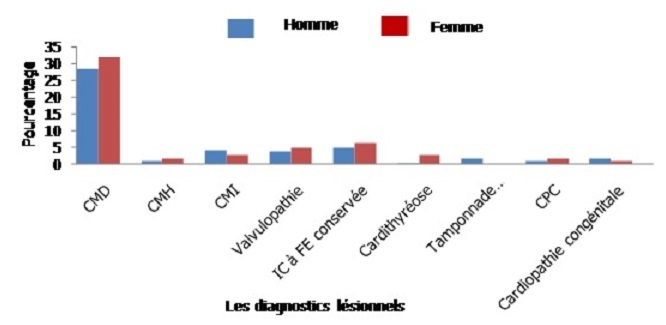
Répartition des patients selon le diagnostic lésionnel de l'insuffisance cardiaque par sexe

La CMD a été la lésion la plus représentée et elle était retrouvée chez 178 patients (60%) avec un sex-ratio H/F de 0,89. La Figure 1 montre la répartition des patients en fonction du diagnostic lésionnel de l'IC. La cardiopathie hypertensive était découverte sous forme de CMD chez 94 patients soit 52,8% des CMD. L'HTA a été retenue comme étiologie de l'IC à fraction d’éjection conservée chez 24 patients (18,75%). Parmi les patients ayant une cardiopathie ischémique, 34 patients (59,6%) avaient une CMD ischémique.

Pour les valvulopathies, 27,6% étaient d'origine rhumatismale. Les péricardites étaient tuberculeuses (4 patients vivants avec le VIH) et néoplasique (1 patient). Au total le VIH était à l'origine des IC chez 10 patients (3,4%) de notre série. Deux patients avaient des séquelles de tuberculose pulmonaire parmi ceux ayant un coeur pulmonaire chronique. Il était difficile de retenir une étiologie formelle dans les autres cas. Les cardiopathies congénitales retrouvées étaient les suivantes: deux persistances du canal artériel, une sténose pulmonaire, deux communications interauriculaires, une communication interventriculaire, une tétralogie de Fallot, et une transposition des gros vaisseaux. Le Tableau 2 montre la répartition des patients en fonction des étiologies des IC.

**Tableau 2:** Répartition des patients selon les étiologies

Etiologies	Effectif	Pourcentage (%)
Cardiopathies hypertensives	128	43,1
Cardiopathie ischémique	57	19,2
Valvulopathies	35	11,8
Cardiomyopathie du péripartum	35	11,8
Cardiomyopathie dilatée primitive	13	4,4
Cardiothyréose	9	3
Myocardite à HIV	6	2
Cardiomyopathie dilatée toxique	6	2
Péricardite	5	1,7
Cœur pulmonaire chronique	8	2,7
Cardiopathies congénitales	8	2,7

## Discussion

La prévalence de l'IC dans notre étude est supérieure à celle retrouvé au Maroc (15%) [9] mais inférieure à celles rapportées dans les autres pays d'Afrique Sub-saharienne. En effet les prévalences étaient les suivantes: Dakar (37,7%) en 2001 [10]; Bamako (41,3%) en 2002 [11]; Congo Brazzaville en 2006 (45,9%) [12], Cameroun (30%) en 2001 [13]. La prédominance féminine de l'HTA, des valvulopathies, des cardiothyréoses et la part exclusive féminine de la CMPP étaient des arguments avancés pour expliquer la prédominance féminine des IC dans notre étude. Le jeune âge de nos patients vient confirmer que l'IC est la principale circonstance de découverte des maladies cardiovasculaires en Afrique. Nos résultats s'expliquent par la recrudescence de l'HTA non ou mal suivie dans une population jeune et l’émergence dans nos sociétés, des facteurs de risque cardiovasculaire tels le diabète, le tabagisme, l'obésité et la dyslipidémie.

La proportion importante de la dyspnée stade III et IV, les oedèmes des membres inférieurs (OMI), les états d'anasarque témoignaient du stade très avancé des IC avant la consultation. Les causes de ces retards de consultation étaient multiples: la pauvreté, l'ignorance, la tradithérapie, les séances de prières et des prises en charge par des personnels para-médicaux avec souvent des traitements non adaptés. Les signes physiques des IC retrouvés dans ce travail étaient identiques à ceux rapportés dans la littérature mais avec des taux statistiques variables d'un auteur à un autre [9, 11–13]. Ces disparités statistiques s'expliquent par le caractère subjectif des examens cliniques d'où la nécessité de faire des examens paracliniques.

La radiographie pulmonaire reste un élément essentiel dans nos régions où l’échodoppler cardiaque est rare et inaccessible à la population. Une cardiomégalie et une stase veineuse pulmonaire plaident en faveur d'une IC. La radiographie pulmonaire permet un diagnostic positif, différentiel, et de retentissement; c'est un élément de suivi. Les signes électrocardiographiques des IC sont moins spécifiques. Les signes radiographiques et électrocardiographiques (cardiomégalie importante, les hypertrophies cavitaires et les troubles de rythmes) étaient rapportés par les études consacrées aux IC en Afrique. Nous partageons avec tous ces auteurs sur le caractère avancé et sévère des anomalies constatées [9–14].

L’échodoppler cardiaque est indispensable pour poser le diagnostic lésionnel des IC. C'est un moyen diagnostic simple, reproductif relativement peu coûté. Mais cet examen n'est pas encore accessible à nos patients. C'est seulement dans notre capitale qu'une échographie cardiaque peut être pratiquée. La conséquence est l'absence ou le retard diagnostique. Les lésions échocardiographiques étaient tellement avancées que l'arme thérapeutique efficace qui restait était la transplantation cardiaque indépendamment des étiologies de ces IC.

La cardiopathie hypertensive était la principale étiologie dans notre série ce qui était conforme aux études en Afrique sub-saharienne [9–14]. Ce qui était regrettable c'est sous la forme de CMD que l'HTA était découverte ou que les patients avaient pris conscience de leur statut d'hypertendu. Un dépistage précoce et la prise en charge adéquate de l'HTA avec les autres facteurs de risque cardiovasculaires sont indispensables pour la réduction des CMD. N'est ce pas un échec des méthodes et moyens de lutte contre l'HTA utilisés en Afrique’ Nous pensons qu'il faut privilégier les campagnes de proximité outre que les médias. On pourra s'appuyer sur les chefferies traditionnelles. On pourra aussi introduire à l’école dès les classes primaires la notion de facteurs de risque cardiovasculaire au même titre que sont enseignées les maladies transmissibles et infectieuses.

La cardiopathie ischémique occupait le deuxième rang des étiologies confirmant la notion de transition épidémiologique en Afrique [4, 15, 16]. Son taux pourrait être plus grand si la coronarographie avait été effectuée chez tous les patients ayant une CMD idiopathique et une CMD hypertensive. Si nous considérons les études réalisées en Afrique sub-saharienne sur les IC, nous pouvons dire que la pathologie ischémique a fait une progression pour se placer en deuxième position [10, 11, 13]. Au Maroc la pathologique ischémique occupe déjà la première place des IC [9].

Les valvulopathies organiques représentaient 8,7% des cas dans notre série. Ce taux est inférieur à ceux de Thiam et Diallo [10, 11] mais était proche du résultat obtenu par Ikama [12]. Dans notre série, les étiologies des valvulopathies étaient dégénératives, rhumatismales, endocarditiques et ischémiques. Nous assistons à une régression des valvulopathies rhumatismales qui font place aux valvulopathies dégénératives et ischémiques. Le recul relatif des valvulopathies rhumatismales est à mettre sous le compte de l'usage répandu des antibiotiques et l'amélioration de l'hygiène de vie [16]. Nous pensons que cette prévalence va continuer par chuter parce que les cas encore rencontrés aujourd'hui sont les séquelles d'un rhumatisme articulaire aigu d'une ou de plusieurs décennies passées. Mais les efforts doivent s'intensifier car la plupart des pays d'Afrique noire ne disposent pas d'infrastructure pour la prise en charge chirurgicale de ces valvulopathies.

Parmi les CMD, le diagnostic positif des CMPP ne pose pas de problème. Il faudrait plus de sensibilisation pour réduire les facteurs de risque et surtout pour que les patientes consultent tôt afin d'améliorer le pronostic. Le diagnostic de CMD éthyliques était posé devant la notion d'alcoolisme chronique, l'absence d'une autre cause d'IC, l'augmentation des triglycérides, des volumes globulaires moyens et des gammas GT. Il s'agit souvent de l'alcool frelaté dont la nature et la teneur de l’éthanol échappent à tout contrôle. Nous avons retrouvé 5, 9% de CMD primitives. Ce taux devrait être plus faible si les moyens d'investigation étaient disponibles tels que la coronarographie, la biopsie myocardique. De même il était difficile de trouver les étiologies des coeurs pulmonaires dans plus de 75% des cas. Nous avons incriminé en partie des séquelles des pathologies infectieuses pulmonaires et une évolution d'une hypertension artérielle pulmonaire primitive.

Enfin notons la part non négligeable des cardiopathies congénitales qui représentaient 2,7% des cas. Il s'agissait de cardiopathies qui devraient être diagnostiquée dès la naissance. Nous insistons sur un bon examen cardiovasculaire des enfants en pédiatrie quelque soit le motif de consultation.

## Conclusion

L'insuffisance cardiaque (IC) est une affection fréquente dans notre pays et en Afrique. Le diagnostic est posé au stade de l'insuffisance cardiaque globale avec des lésions sévères et évoluées. La population concernée était jeune. L'HTA, les coronaropathies, la cardiomyopathie du péripartum, les valvulopathies, les cardiomyopathies toxiques et infectieuses en étaient les principales étiologies. Nous assistons à un recul des valvulopathies rhumatismales. Des mesures doivent être prises pour la prévention et la correction des facteurs de risque cardiovasculaire que sont l'HTA, le diabète, le tabagisme, la dyslipidémie, l'obésité, la sédentarité et le stress. Cette prévention passe aussi par la réduction de l'alcoolisme et la lutte contre les maladies infectieuses telles que le VIH-SIDA.
